# Construction of chitosan-supported nickel cobaltite composite for efficient electrochemical capacitor and water-splitting applications

**DOI:** 10.1038/s41598-023-49692-z

**Published:** 2024-01-30

**Authors:** Shymaa S. Medany, Ayman Nafady, Razium Ali Soomro, Mahmoud A. Hefnawy

**Affiliations:** 1 Chemistry Department, Faculty of Science, Cairo University, 12613, Giza, Egypt; 2 Department of Chemistry, College of Science, King Saud University, 11451 Riyadh, Saudi Arabia; 3 State Key Laboratory of Chemical Resource Engineering School of Chemistry, Beijing Advanced Innovation Center for Soft Matter Science and Engineering, Beijing, 100029 People’s Republic of China

**Keywords:** Electrocatalysis, Electrochemistry, Fuel cells

## Abstract

The construction of highly efficient electrode material is of considerable interest, particularly for high capacitance and water-splitting applications. Herein, we present the preparation of a NiCo_2_O_4_-Chitosan (NC@Chit) nanocomposite using a simple hydrothermal technique designed for applications in high capacitance and water-splitting. The structure/composition of the NC@Chit composite was characterized using different analytical methods, containing electron microscope (SEM and TEM), and powder X-ray diffraction (XRD). When configured as an anode material, the NC@Chit displayed a high capacitance of 234 and 345 F g^−1^ (@1Ag^−1^ for GC/NC and NC@Chit, respectively) in an alkaline electrolyte. The direct use of the catalyst in electrocatalytic water-splitting i.e., HER and OER achieved an overpotential of 240 mV and 310 mV at a current density of 10 mA cm^−2^, respectively. The obtained Tafel slopes for OER and HER were 62 and 71 mV dec^−1^, respectively whereas the stability and durability of the fabricated electrodes were assessed through prolonged chronoamperometry measurement at constant for 10 h. The electrochemical water splitting was studied for modified nickel cobaltite surface using an impedance tool, and the charge transfer resistances were utilized to estimate the electrode activity.

## Introduction

Population expansion and socioeconomic advancement have contributed to a sharp rise in the world's energy demands. Nevertheless, the excessive reliance on conventional fossil fuels has resulted in rapid resource depletion and environmental contamination challenges^1,2^. Creating innovative, clean, and renewable energy sources is highly desirable. The advantages of hydrogen energy, such as its cleanliness, sustainability, and high energy density, are well-established^3–6^. Water electrolysis, as a method for hydrogen production, is particularly noteworthy for its potential to yield high-purity hydrogen, with no carbon emissions and environmental friendliness^7–9^. In the context of catalysts, the abundance of materials, low cost of synthesis, and advantageous catalytic characteristics of transition-metal-based (TM-based) catalysts have drawn much attention than many types of catalysts^10–14^. Currently, platinum (Pt) or Pt-based materials remain the most effective electrocatalysts for the hydrogen evolution reaction (HER) due to their suitable adsorption energy with intermediates. However, their widespread implementation is hindered by their high cost and scarcity, as evidenced by previous research^15–17^. While effective electrocatalysts have traditionally been synthesized using precious metals like Pt, Ir, and Ru, recent advancements have introduced alternatives based on non-noble and earth-abundant metals (such as metal alloys^18,19^, metal carbides, and nitrides^20^, and molecular metal complexes^21^). For energy-related devices, nickel, its alloys (NiCo^18,22^, NiMo^23^, NiFe^24–26^, and NiMn^27,28^), or the equivalent metal oxides represent a promising family of non-precious metal catalysts.

The chemical composition of XY_2_O_4_ characterizes metal oxides with a spinel structure where Y represents a trivalent metal ion at the octahedral position, and X represents a divalent metal ion at the tetrahedral position, while oxygen anions are arranged in a close-packed cubic lattice^29^. Until now, c spinel oxides materials such as CuBi_2_O_4_^30^, CuFe_2_O_4_^31^, ZnFe_2_O_4_^32^, and NiCo_2_O_4_^33,34^ have been explored for thier structure–activity relationship garnering significant attention to water splitting application.

At present, the predominant electrode materials utilized in commercial supercapacitors consist of electric double-layer capacitive materials primarily composed of pure carbon^35,36^. Typically, these devices exhibit favorable cycle longevity and notable maximum power density. However, their specific capacitance and energy density must meet the escalating demand for peak-power augmentation in electric vehicles^37^. When comparing pseudocapacitive materials to pure carbon-based materials, it becomes evident that the former can significantly augment supercapacitors' specific capacitance and energy density. This enhancement is achieved through the utilization of interfacial reversible faradaic reactions as a means to store energy^38^. Furthermore, integrating pseudocapacitive materials with electric double-layer capacitive materials in constructing asymmetric supercapacitors can lead to an additional improvement in the energy and power density of these supercapacitors^39,40^. Traditional pseudocapacitive materials primarily consist of common transitional metal oxides/hydroxides, such as ruthenium dioxide (RuO_2_), manganese dioxide (MnO_2_), nickel oxide (NiO), nickel hydroxide (Ni(OH)_2_), cobalt oxide (Co_3_O_4_), cobalt hydroxide (Co(OH)_2_), iron oxide (Fe_3_O_4_), and their binary counterparts^41–44^.

NiCo_2_O_4_, among various metal oxides, exhibits advantageous characteristics such as affordability, minimal toxicity, and widespread availability. Additionally, the NiCo_2_O_4_ could be hybridized with variety of diverse materials to achieve enhanced supercapacitive properties^45,46^.

**Figure 1:**
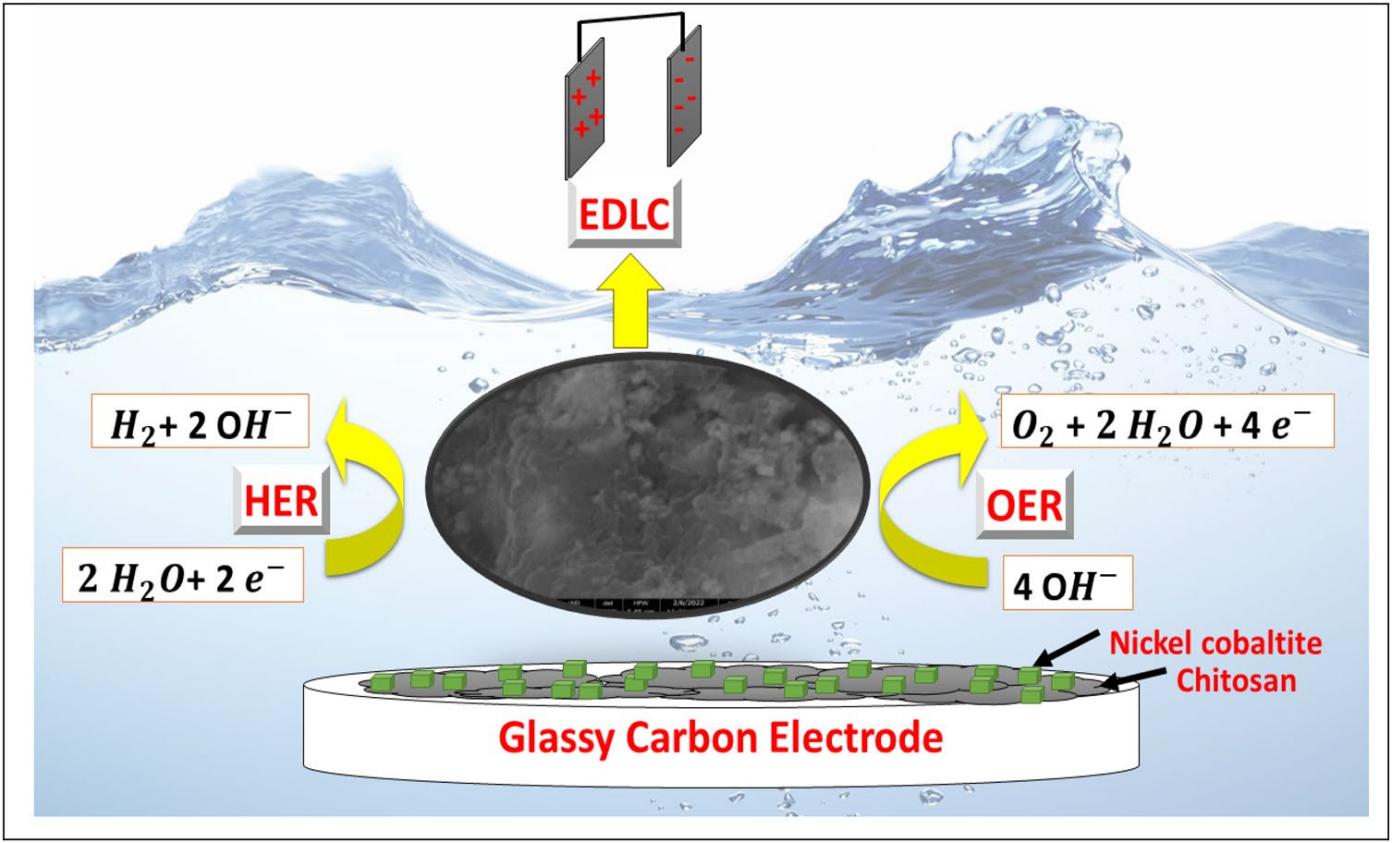
Illustration of modified glassy carbon surface for different energy storage applications.

In this study, a composite of nickel cobaltite was synthesized using a hydrothermal technique. The resulting modified glassy carbon electrode, with the nickel cobaltite-chitosan composite (GC/NC@Chit), was utilized for both capacitance and water-splitting applications (see Fig. 1). The prepared materials were characterized using several analytical techniques, while electrochemical techniques such as galvanostatic charging discharging (GCD), and electrochemical impedance (EIS) were used to measure capacitance behavior. The activity of the electrodes toward water splitting was investigated using voltammetry, constant potential chronoamperometry, and constant current potentiometry.

## Experimental section

### Preparation of nickel cobaltite

All chemicals utilized in the study were commercially available and employed without further modification. During the standard synthesis process, urea (3.1 g), Ni(CH_3_COO)_2_·4H_2_O, and Co(CH_3_COO)_2_·4H_2_O were added to the solution, with a combined weight of nickel and cobalt acetates amounting to 1.28 g (approximately 4.40 mmol). The solution was agitated for an additional duration of 20 min. Subsequently, the solution was introduced into a stainless-steel autoclave lined with Teflon and subjected to a temperature of 150 °C for 6 h. Upon cooling the autoclave to ambient temperature, the precursor underwent filtration and was subjected to a thorough washing with deionized water, followed by drying in an oven. Subsequently, the desiccated specimens underwent a calcination process at a temperature of 500 °C for 3 h without any inert gas^47^.

### Preparation of nickel cobaltite-supported chitosan (NC@Chit)

NC@Chit composite was produced by homogenizing chitosan with nickel cobaltite nanoparticles as follows: In a standard procedure, 0.5 g of chitosan was placed in a 250 mL beaker, and the temperature was gradually increased with continuous stirring. Subsequently, 0.5 g of NiCo_2_O_4_ was added to the mixture. The solution temperature was then lowered to room temperature. The addition of nickel cobaltite to the chitosan solution led to the bridging and encapsulation of nickel cobaltite nanoparticles. To achieve a thick consistency, 1 mL of 10% acetic acid was introduced into the mixture and agitated until the desired solution consistency was attained. Following a duration of 10 min, the liquid was filtrated and subsequently rinsed with double distilled water. The product was dried at 60 °C overnight before using as a catalyst.

### Electrode preparation

The working electrode employed in the experiment was a glassy carbon electrode with a 3 mm diameter corresponding to an area of 0.0707 cm^2^. Initially, the surface underwent a polishing process utilizing a delicate emery paper, followed by a thorough cleansing procedure involving ethanol and isopropanol. The casting suspension was prepared by suspended 10 mg of the electrocatalyst powder (NC or NC@Chit) in a mixture of 250 µL of ethanol with 250 µL of 5 wt% Nafion sonicated for 30 min. The electrode was constructed following the deposition of 40 µL catalyst ink onto the GCE surface followed by drying at 50 °C for 4 h. The electrochemical experiments were conducted using the Autolab PGSTAT128N instrument, and the NOVA software facilitated the impedance spectrum analysis. The setup included an Ag/AgCl/KCl (sat.) reference electrode and Pt wire as the counter electrode. The working electrodes employed in the experiment were GC/NC and GC/NC@Chit. For error bar, the data was repeated three times to find out standard error. All potential values were referenced against Ag/AgCl/sat KCl, and for hydrogen evolution reaction (HER) applications, the potential was corrected to the reversible hydrogen electrode (RHE). The potential was determined with respect to the reversible hydrogen electrode (RHE) in relation to the subsequent equations^48^:

1 $$ {\text{E}}_{{{\text{RHE}}}} = {\text{E}}_{{{\text{Ag}}/{\text{AgCl}}}} + {\text{E}}^\circ_{{{\text{Ag}}/{\text{AgCl}}}} + 0.0{\text{59 pH}} $$

The electrochemical experiments were conducted in 1.0 M solution of KOH as the supporting electrolyte. The potential was standardized by employing a reversible hydrogen electrode.

2 $$ {\text{E}}_{{{\text{RHE}}}} = {\text{E}}_{{{\text{Ag}}/{\text{AgCl}}}} + {1}.0{23}\left( {{1}.0\,{\text{M}}\,{\text{NaOH}}\sim {\text{pH}} = {14}} \right)\& \left( {{\text{E}}^\circ_{{{\text{Ag}}/{\text{AgCl}}}} = 0.{197}\,{\text{V}}} \right) $$

## Result and discussion

### Structure and surface analyses

**Figure 2:**
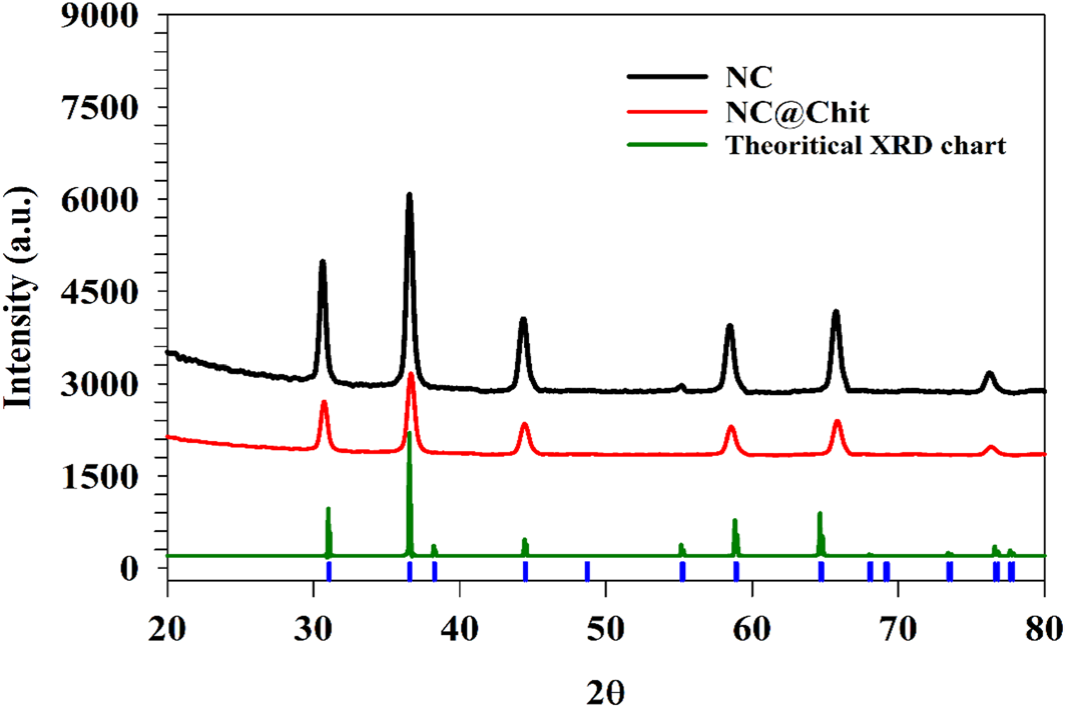
XRD pattern of NC@Chit samples referenced against standard peaks of NiCo_2_O_4_ crystal.

The chemical structure of prepared chitosan-based nickel cobaltite was characterized using powder X-ray diffraction. Figure 2 shows the X-ray diffraction (XRD) pattern with diffraction peaks for crystalline NC@Chit referenced against amorphous peaks of m-C (JCPDS no. 20-0781)^49^ confirming the spinel NiCo_2_O_4_ structure with hkl planes of 220, 311, 400, 511, 440, and 533 for peaks at 2θ equal to 30.4, 36.5, 44.1, 58.1, 65.2, and 76.1, respectively. The hump observed at 2θ ~ 12.6 was attributed to the chitosan sheets^50,51^.

The observed peak intensity in the sample containing chitosan decreased, attributed to the presence of embedded nanoparticles within the chitosan sheets. The interaction between chitosan and nickel cobaltite resulted in a modification of the lattice structure^52–54^. To comprehensively understand the molecular-level processes, XRD pattern of NiCo_2_O_4_ was referenced against standard peaks of NiCo_2_O_4_ crystal as illustrated in Fig. 2. The close match between the theoretical and experimental XRD results confirmed the high quality of the prepared modified NiCo_2_O_4_ material using Material studio software.

**Figure 3:**
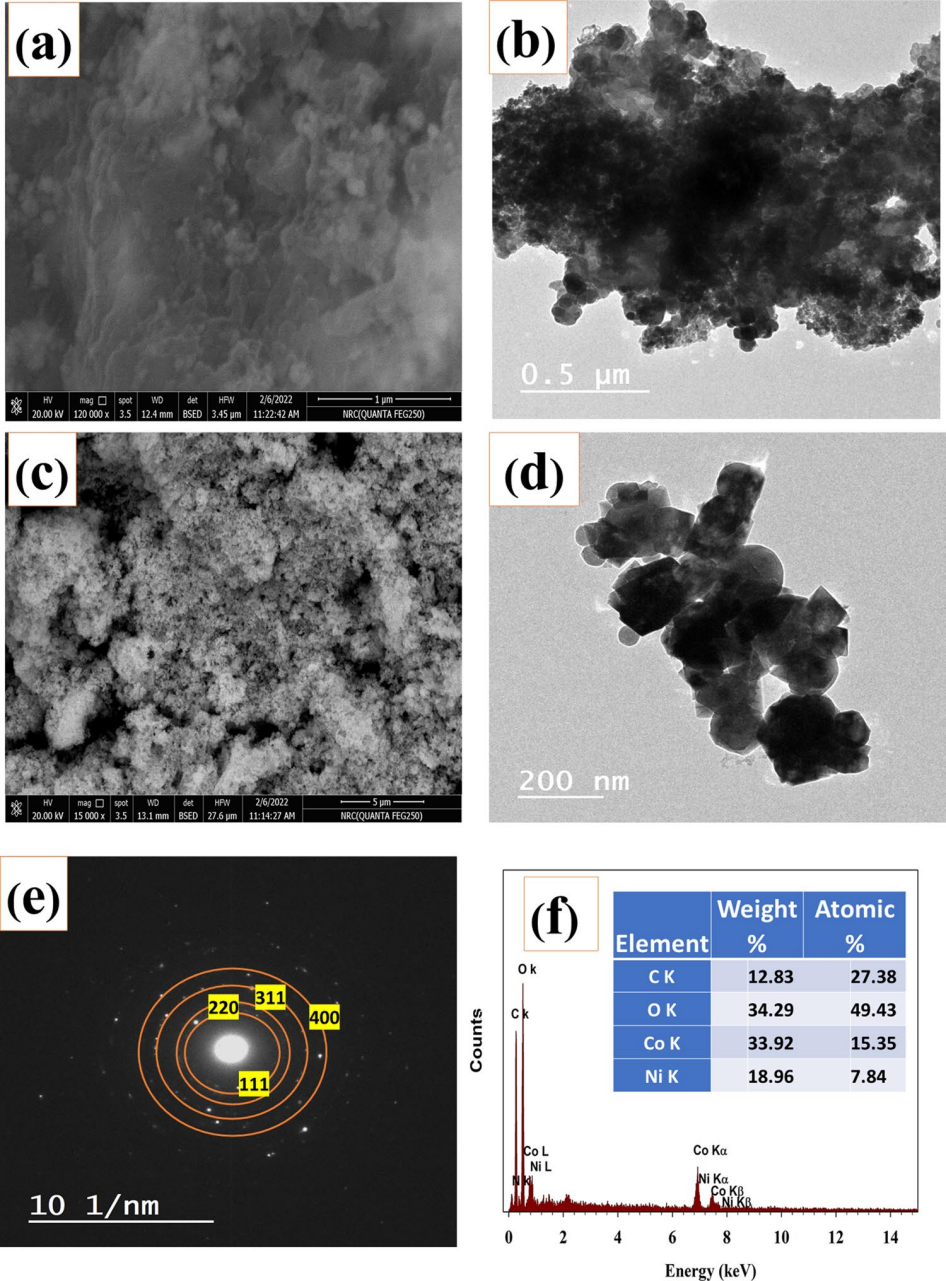
Surface characterization of NC@Chit sample by (**a**) SEM of Chitosan modified NiCo_2_O_4_, (**b**) TEM Chitosan modified NiCo_2_O_4_, (**c**) SEM of pristine NiCo_2_O_4_, (**d**) TEM of pristine NiCo_2_O_4_, (**e**) diffraction, (**f**) EDX spectrum.

Scanning electron microscopy (SEM) was utilized to examine the morphological characteristics of the nickel cobaltite nanoparticles, as depicted in Fig. 3a. The particles exhibited a size distribution within the 35–80 nm range. The diminutive size of nickel cobaltite particles suggested an augmented activity level in the synthesized substances. The incorporation of nickel cobaltite into the chitosan sheets is illustrated in Fig. 3a, showcasing well-distributed nickel cobaltite particles on the chitosan sheets. Furthermore, the inclusion of chitosan has been observed to enhance water splitting, a phenomenon confirmed by subsequent electrochemical experiments.

Additionally, TEM was employed to determine the morphology of nickel cobaltite nanoparticles. Figure 3b shows the TEM images of NC@Chit composites with mean particle diameter estimated to be around 20–50 nm. The study unveiled that the nanospheres composed of NiCo_2_O_4_ exhibited attachment to the chitosan sheets. The presence of NC on the chitosan sheet is validated by utilizing the corresponding transmission electron microscopy (TEM) diffraction patterns. For comparison, pristine nickel cobaltite was characterized using SEM, as depicted in Fig. 3c, revealing nanoparticles with a particle size of approximately 30 nm. Additionally, the TEM of pristine nickel cobaltite was employed in Fig. 3d, highlighting the cubic structure of the particles. The Miller indices (Fig. 3e) from the observed SEAD pattern represents (400), (311), (220), and (111) planes, respectively whereas the EDX spectra confirmed the of Ni, Co, O, C, and N elements as major constituents of NC@Chit (Fig. 3f). The elemental proportions depicted in the inset figure correspond to the intended composition of NC, wherein the ratio of Ni to Co is 1:2.

### Electrochemical capacitor

Cyclic voltammetry (CV) was employed to assess the activity of modified GC/NC and GC/NC@Chit in a 1.0 M KOH solution. The activation process, a pivotal step in water-splitting reactions involving nickel-based electrodes, enhanced the performance of the electrodes. This procedure led to the formation of NiOOH, exhibiting high electrocatalytic activity. The activation process was carried out CV at a scan rate of 50 mV s^−1^ for 200 cycles. A rise in current is caused by the NiOOH production event throughout subsequent cycles. The thickness of the NiOOH layer increases in direct proportion to the number of potential sweeps. The presence of OH^-^ ions can be ascribed to, in accordance with the subsequent equation^55–57^:

3 $$ 6{\text{NiOOH}} + 6{\text{H}}_{2} {\text{O}} + 6e^{ - } \leftrightarrow 6{\text{Ni(OH)}}_{2} + 6{\text{OH}}^{ - } $$

**Figure 4:**
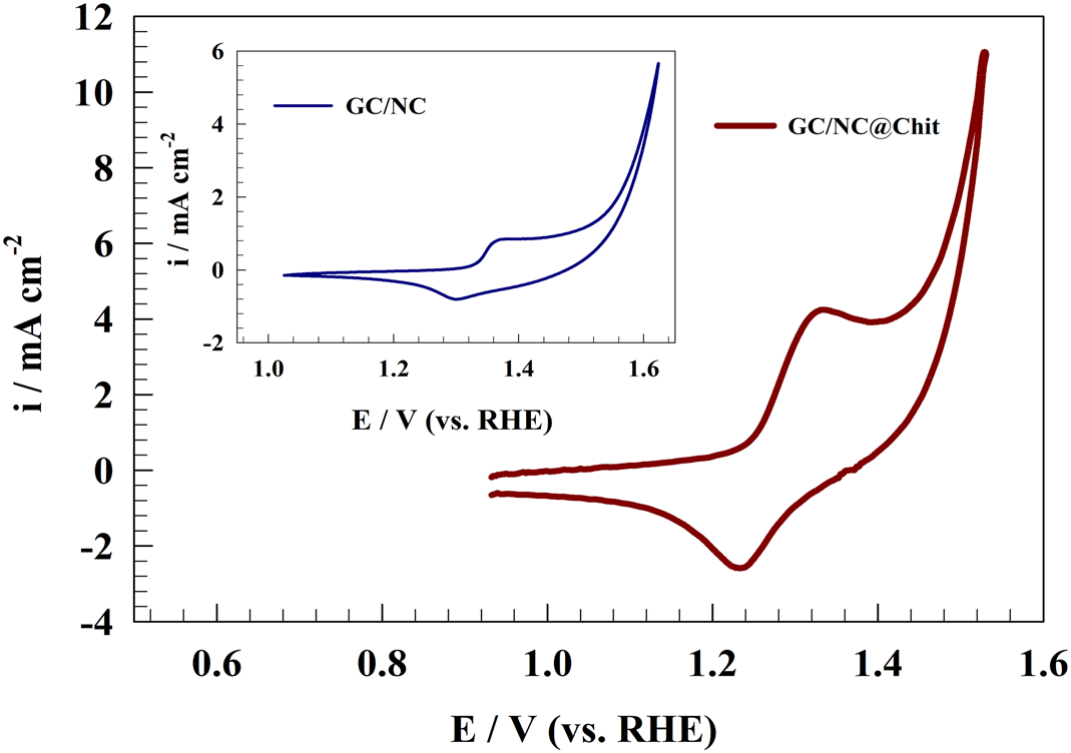
CV of modified GC/NC@Chit electrode in a alkaline solution.

Figure 4 shows the CV of modified GC/NC@Chit electrode in 1.0 M NaOH with corresponding redox peak within the specified potential window (1.2–1.35 V (vs. RHE)) attributed to the conversion of Ni(II) to Ni(III) and vice versa.

**Figure 5:**
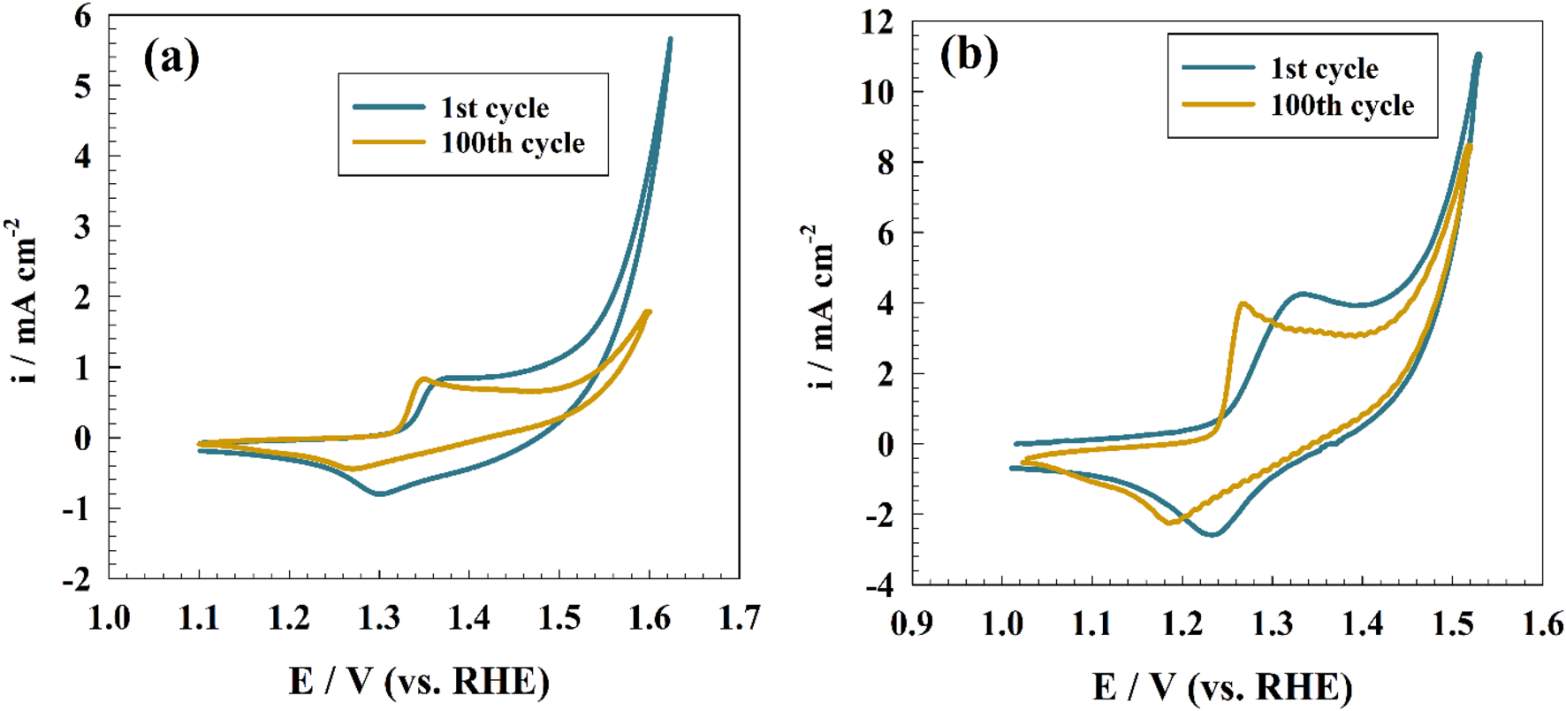
Stability test for modified (**a**) GC/NC and (**b**) GC/NC@Chit electrodes in KOH.

Furthermore, the stability of redox species formation for Ni-based electrode was studied using repeated CV of the modified GC/NC and GC/NC@Chit in alkaline medium for 100 cycles. However, the increase in number of CVs in KOH lead to increase the thickness of the Ni(OH)_2_/NiOOH layer that responsible for electrode activity. On the other hand, other factors could decrease the electrode current such as surface stability or poisoning. As shown in Fig. 5, the CVs for both GC/NC and GC/NC@Chit before and after 100 cycles in KOH. For GC/NC, the slight shift in peak position due to the increase in thickness owing to conversion of more species to higher electroactive NiOOH that make the process more thermodynamic favored. Else, GC/NC@Chit showed higher current stability along due to the presence of chitosan substrate which enhance the stability of nickel cobaltite on electrode surface.

**Figure 6:**
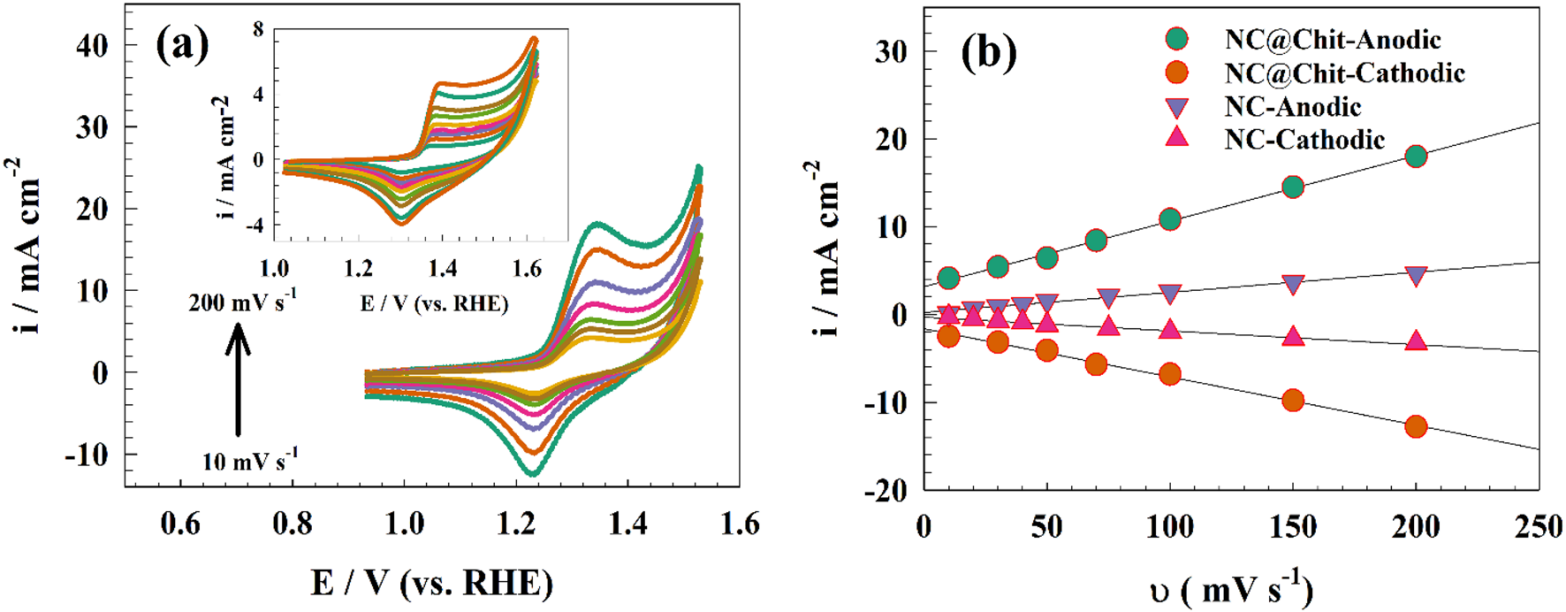
(**a**) CVs of GC/NC@Chit modified electrode at different sweep rates (10–200 mV s^−1^), with inset figure showing CVs of GC/NC electrode at various sweep rates (10–200 mV s^−1^). (**b**) anodic current vs. scan rate for both GC/NC@Chit and GC/NC electrodes.

The CV responses were recorded against different scan rates to confirm the kinetics of the redox process. Figure 6a shows the CV curves of modified GC/NC@Chit electrode at scan rates extending from 10 to 200 mV s^−1^ with inset figure depicting the linearity of the increasing current response with scan rate. The surface coverage of the NiOOH active species can be calculated using the following equation:

4 $$ {\text{i}} = \left( {\frac{{{\text{n}}^{2} F^{2} }}{4RT}} \right)\upsilon {\text{A}}\Gamma $$

where i is redox current, n = 1 (number of electrons), A = 0.0707 cm^2^ (surface area), Γ (surface coverage), and υ (scan rate).

Consequently, surface coverage was estimated from the linear relation between anodic current vs. scan rate (see Fig. 6b). The calculated surface coverage (Γ) for NC@Chit and /NC are 1.67 $\times$ ${10}^{-9}$ and 8.61 $\times$ ${10}^{-10}$ mol cm^−2^, respectively.

**Figure 7:**
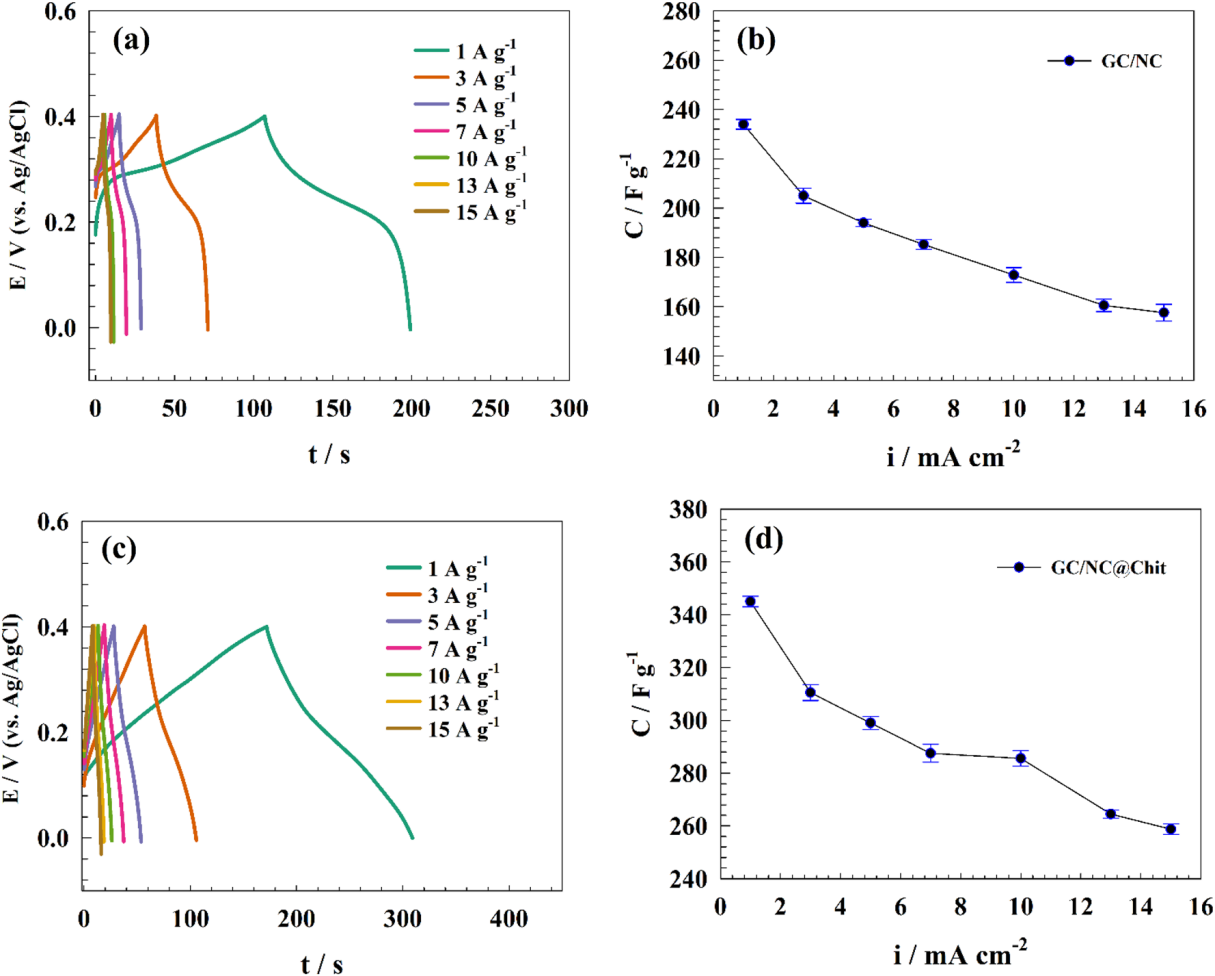
(**a**) GCD of GC/NC electrode, (**b**) Relation between capacitance versus applied current for GC/NC electrode, (**c**) GCD of GC/NC@Chit electrode, (**d**) Relation between capacitance versus applied current for GC/NC@Chit electrode.

The NC and NC@Chit composites display uniform distribution across the chitosan sheets, showcasing effective areas and favorable hydrophilic properties. As a result, the GC/NC@Chit composite exhibits a high specific capacitance (Cs) value. Figure 7a,c illustrates the GCD curve of the prepared active material, measured at different current densities with pristine nickel cobaltite exhibiting battery-like curve with capacitance (see Fig. 7a). For modified GC/NC electrode, the capacitance value obtained were 234, 205, 194, 185, 173, 160, and 157 F g^−1^ at different current densities of 1, 3, 7, 10, 13, and 15 A g^−1^ respectively (see Fig. 7b). On the other hand, spinel oxide incorporation in chitosan sheets exhibited high capacitance of 345, 310, 299, 287, 285, 264, and 258 F g^−1^ at different current densities of 1, 3, 7, 10, 13, and 15 A g^−1^ respectively (see Fig. 7d).

**Figure 8:**
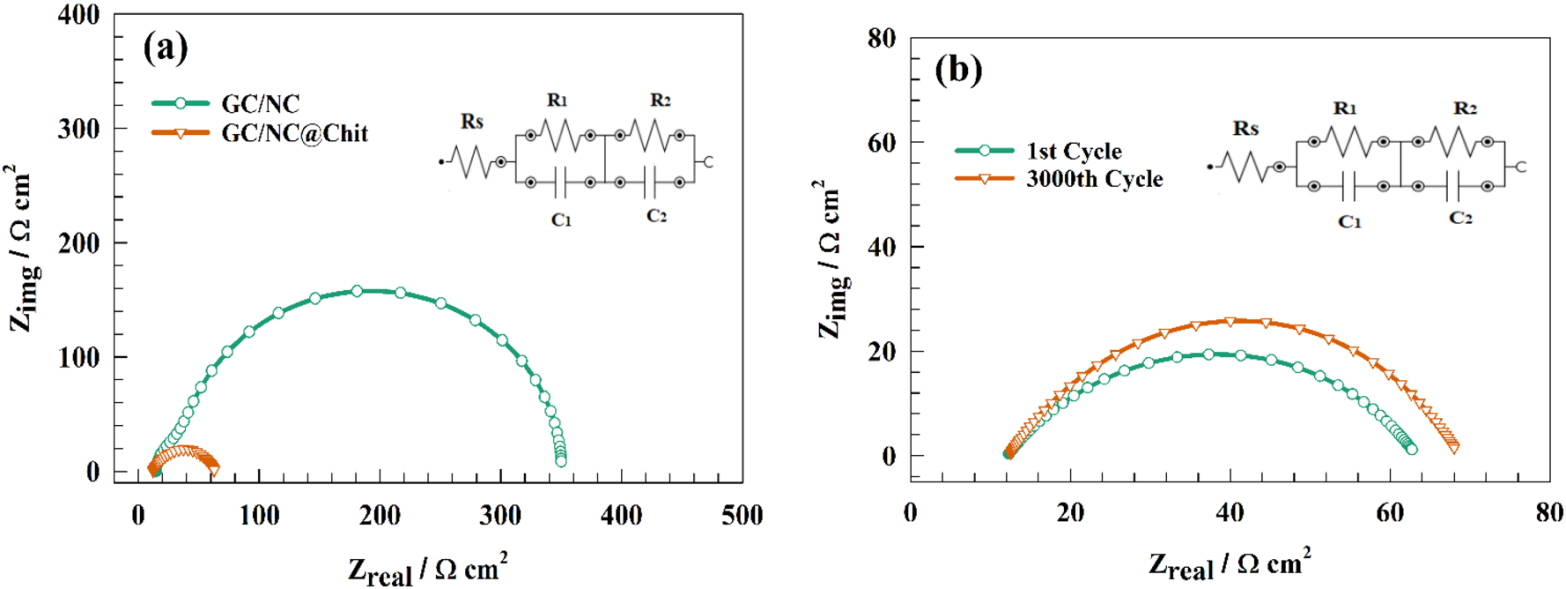
(**a**) Representation of EIS plot of NC and NC@Chit surfaces at constant potential equal 1.4 V. (**b**) Nyquist plot of the modified GC/NC@Chit after 3000th GCD cycles.

Electrochemical impedance spectroscopy (EIS) measurements were utilized on both the unmodified and modified NC in order to elucidate the performance of the chitosan-based surface compared with the pristine GC/NC electrode. Figure 8a shows the Nyquist plot for NC and NC@Chit electrodes in the alkaline medium in a solution of 1.0 M KOH at a constant potential of 1.4 V (RHE). The presence of a semi-circuit could be attributed to the charge transfer process. Whereas, the lower diameter of the GC/NC@Chit electrode compared to the unmodified NC electrode counterparts represents higher activity of the chitosan-based electrode for Ni(II)/Ni(III) conversion. The equivalent circuit fitting is shown as inset of Fig. 8, where the tabulated values of R_s_ (solution resistance) connected to R_1_, and R_2_ (charge transfer resistances for outer and inner layer) of the competitive materials are provided in Table 1.

**Table 1:** EIS fitting parameters for Nyquist plot of different modified surfaces.

Electrode	R_s_ (Ω cm^2^)	R_1_ (Ω cm^2^)	C_1_ (F)	R_2_ (Ω cm^2^)	C_2_ (F)
GC/NC	14.8	24.9	0.00068616	360.2	0.00013797
GC/NC@Chit	12.1	21.8	0.00142678	71.3	0.0012675

Figure 8b shows EIS of the GC/NC@Chit after stability test. Whereas, shift in solution resistance (Rs) was observed after stability test. Otherwise, the charge transfer shifted to higher value because of the decrease of surface electrochemical activity.

**Figure 9:**
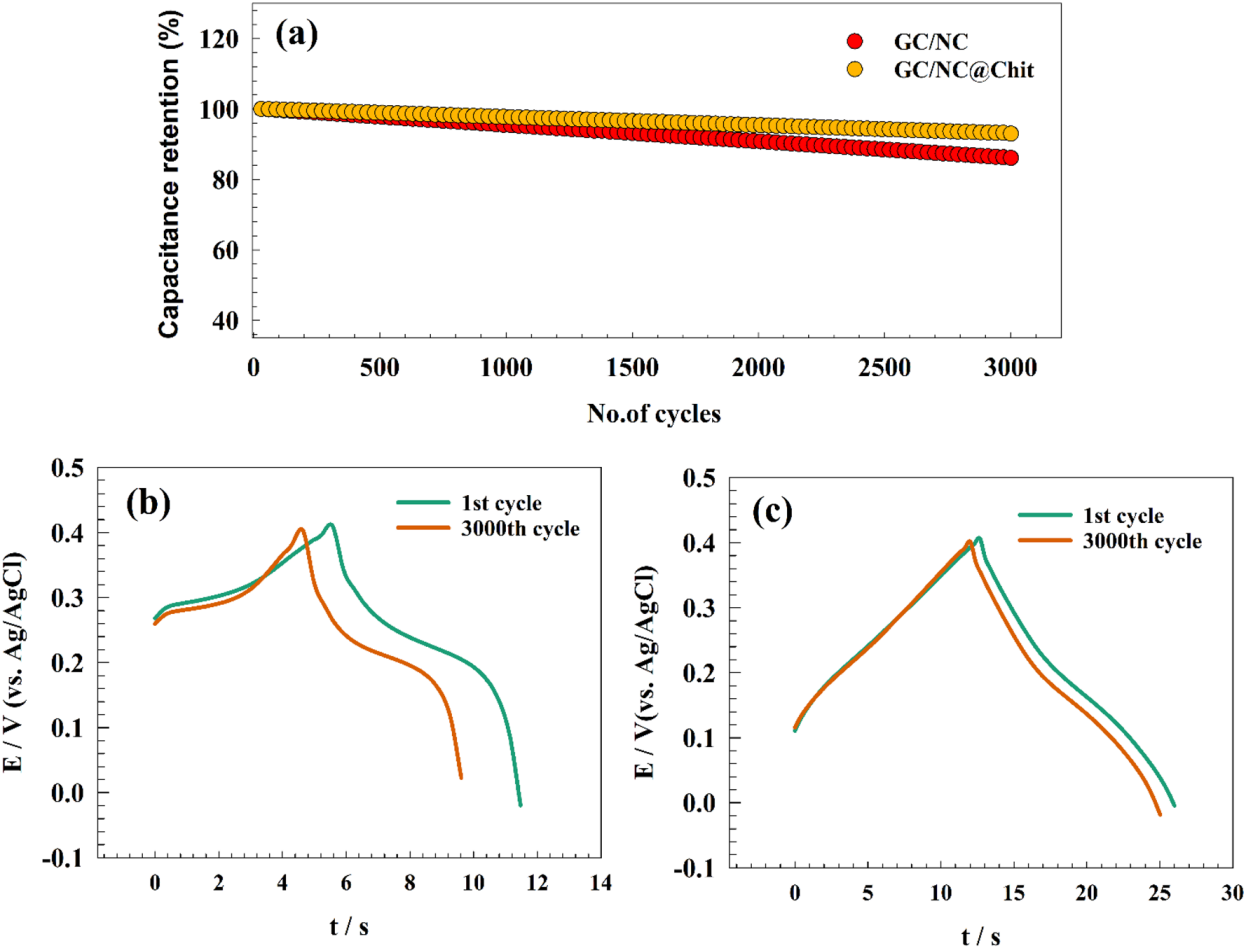
(**a**) Repeated GCD of modified GC/NC, and GC/NC@Chit, Comparison between 1st versus 3000th cycles for (**b**) GC/NC, and (**c**) GC/NC@Chit.

The stability test is a crucial parameter for evaluating the performance of supercapacitor materials. However, both NC and NC@Chit composites exhibited remarkable stability characteristics. Figure 9a illustrates the stability of both modified GC NC and GC/NC@Chit electrodes under a current density of 10 A g^−1^ throughout 3000 cycles. The GC/NC composite exhibited a capacitive retention of 86% after undergoing 3000 cycles, demonstrating a high level of stability. Additionally, GC/NC@Chit electrode demonstrated an impressive capacitive retention of 93% after continuous cycling. The remarkable stability of the GC/NC@Chit composite can be attributed to the robust interaction among nickel cobaltite, chitosan, and the electrode surface, as well as the strong interlayer interaction among the chitosan sheets. Furthermore, based on the data presented in Fig. 9b,c, differences in capacitance and IR drop before and after stability can be observed in the GCD curve.

### Water splitting application

The oxygen evolution process holds significant importance in the functioning of fuel cells and batteries, as it facilitates the conversion of chemical energy to electrical energy^58,59^. Various electrochemical methods have been utilized to determine the mechanism of the oxygen evolution reaction.

One of the prevalent pathways for the electrochemical conversion of hydroxide to molecular oxygen involves a two-step electrochemical process.

The initial step involved the adsorption of hydroxide ions onto the electrode surface, resulting in the formation of hydroxide adsorbates. Subsequently, the adsorbed hydroxide species interacted with hydroxide ions present in the surrounding medium, producing adsorbed oxygen species. The ultimate stage involves the discharge of adsorbed atomic oxygen, resulting in the production of molecular oxygen.

The operational framework of oxygen evolution reactions (OER) can be established in the following manner^60^:

5 $$ {\text{NC}} + {\text{OH}}^{ - } \leftrightarrow \left( {{\text{NC}}} \right){\text{OH}} + {\text{e}}^{ - } $$

6 $$ \left( {{\text{NC}}} \right){\text{OH}} + {\text{OH}}^{ - } \leftrightarrow \left( {{\text{NC}}} \right){\text{O}}^{ - } + {\text{H}}_{{2}} {\text{O}} $$

7 $$ \left( {{\text{NC}}} \right){\text{O}}^{ - } \leftrightarrow \left( {{\text{NC}}} \right){\text{O}} + {\text{e}}^{ - } $$

8 $$ {2}\left( {{\text{NC}}} \right){\text{O}} \leftrightarrow {2}\left( {{\text{NC}}} \right) + {\text{O}}_{{2}} + {\text{2e}}^{ - } $$

**Figure 10:**
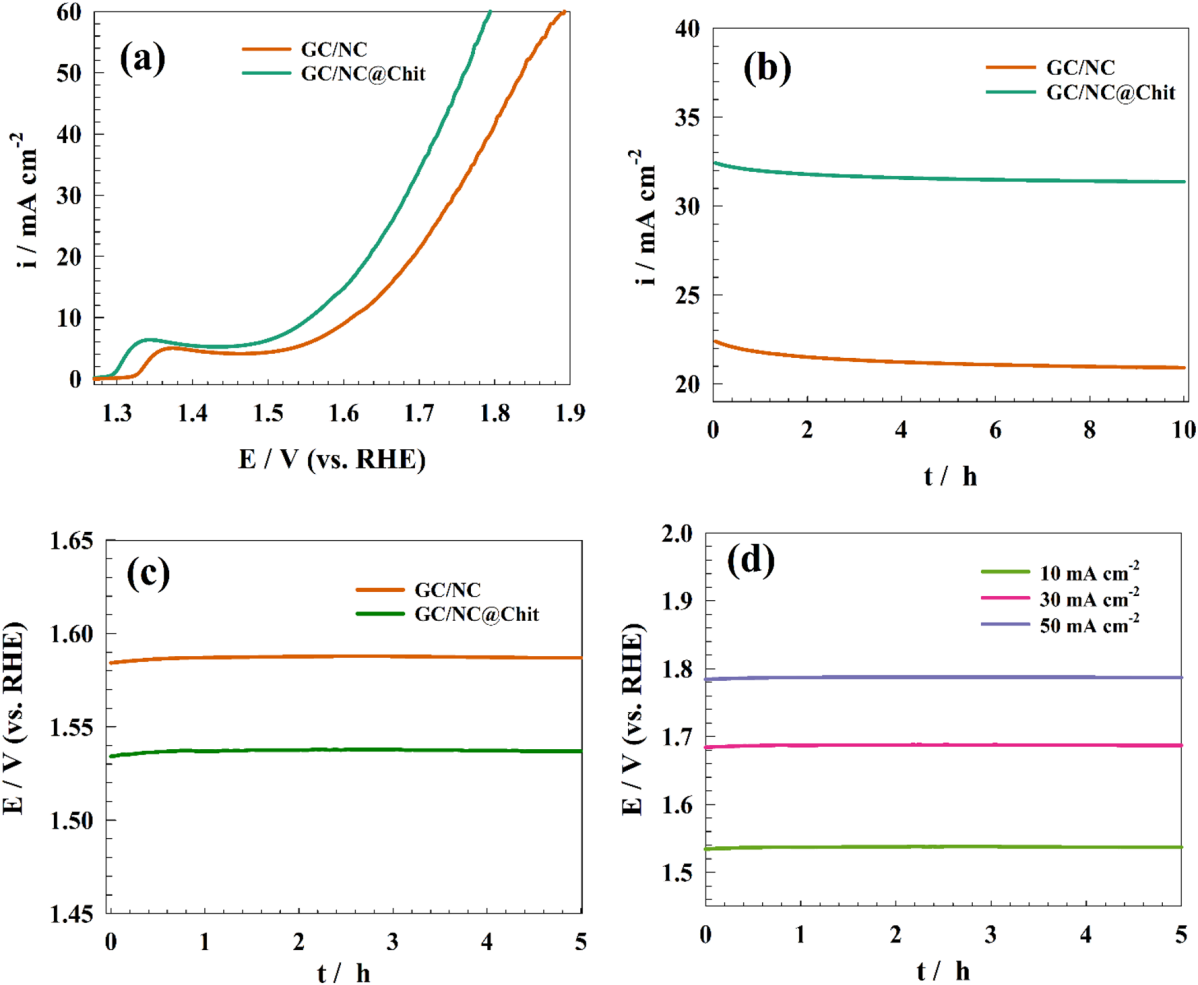
(**a**) LSV of the NC and NC@Chit for OER, (**b**) Chronoamperogram for OER over different surfaces, (**c**) Chronopotentiogram of different modified surfaces for OER, (**d**) Chronopotentiogram of modified GC/NC@Chit at different constant current densities.

Figure 10a depicts the OER (oxygen evolution reaction) on the GC/NC and GC/NC@Chit under the influence of 1.0 M NaOH. As per the analysis, a single oxidation peak can be attributed to converting Ni(II) to Ni(III) where the GC/NC@Chit exhibited a notable current density for oxygen evolution reaction (OER) at a comparatively reduced potential than its GC/NC equivalent.

Furthermore, the electrode's durability in the OER holds significance in electrochemical splitting of water. Chronoamperometry was used to examine the sustained durability of the electrode concerning the (OER). Figure 10b illustrates the chronoamperogram of the GC/NC and GC/NC@Chit modified electrodes in a 1.0 M NaOH solution, with a consistent potential of 1.7 V (versus RHE). The current density of GC/NC and GC/NC@Chit demonstrated a corresponding reduction in its initial value by 8.4 and 4.7%. The current reduction presently observed is attributed to the mechanical corrosion of the electrocatalyst’s surface caused by the evolved gases passing through. Additionally, the electrodes' enhanced long-term durability towards OER is demonstrated by the slight fluctuations in the oxidation current.

In addition, the electrode's galvanostatic endurance was examined for OER. The constant potential chronopotentiometry for two modified surfaces, GC/NC and GC/NC@Chit, is shown in Fig. 10c at a current of 10 mA cm^−2^. As seen, the electrode's overpotential (ƞ) reached 440 and 310 mV, respectively which could be ascribed to superior OER capability of the chitosan-based electrode. Additionally, modified GC/NC@Chit was tested for galvanostatic stability at varied current densities, including 10, 30, and 50 mA cm^−2^, in a solution of 1.0 M NaOH. While at an overpotential of 310, 470, and 630 mV for a current density of 10, 30, and 50 mA cm^−2^ (see Fig. 10d). A comparative evaluation of the NC@Chit electrode's performance for oxygen evolution reaction (OER) in relation to other composites is provided in Table 2.

**Table 2:** Comparative evaluation GC/NC@Chit electrode and other modified electrodes mentioned in the literature for OER.

Surface	ƞ (mV)@10 mA cm^–2^	Tafel slope (mV dec^–1^)	Electrolyte	References
GC/NC@Chit	310	67	1.0 M NaOH	This work
NiCo nanosheets	332	41	1.0 M KOH	^61^
1D- NiCo_2_O_4_	260	104	1.0 M KOH	^62^
GC/Co_3_O_4_	270	64	1.0 M KOH	^63^
GC/LiCoO_2_	430	48	1.0 M KOH	^64^
GC/NiCo-LDH	335	41	1.0 M KOH	^65^
GC/NiFe_2_O_4_	440	98	0.1 M KOH	^66^
PdO@CoNi_2_S_4_	230	72	1.0 M KOH	^67^
PdONPs@Co_3_O_4_	250	58	0.1 M KOH	^68^

**Figure 11:**
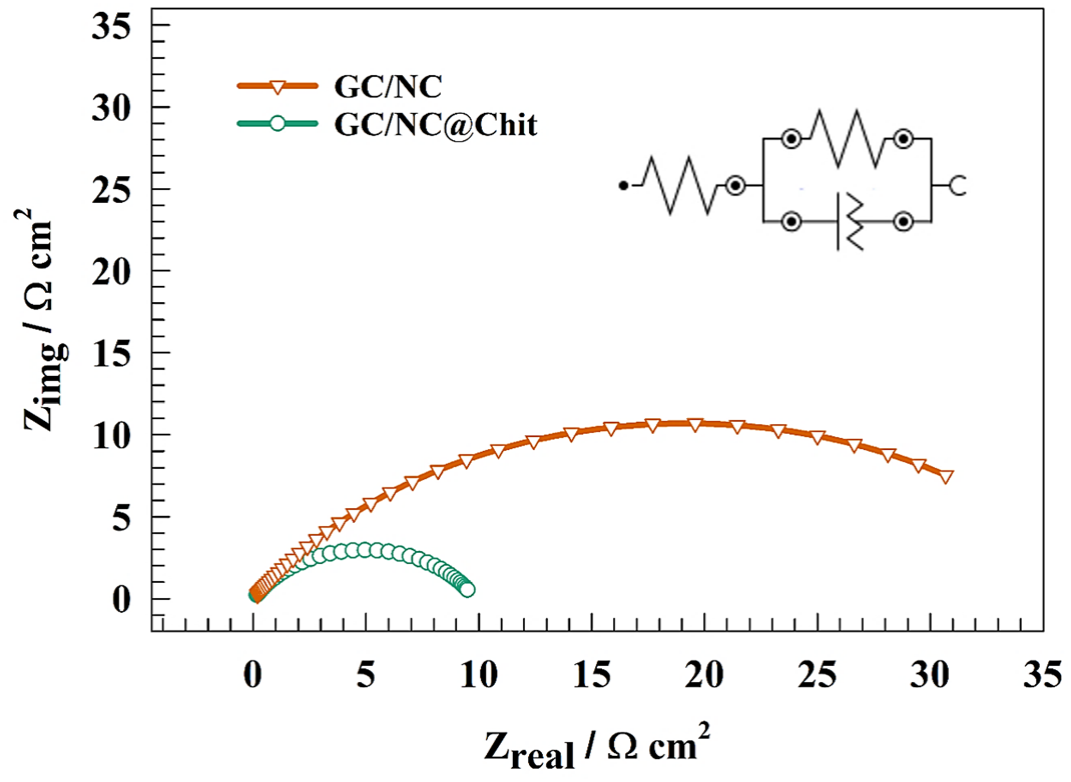
Representation of EIS plot of NC and NC@Chit surfaces at constant potential for OER.

The charge transfer resistance across different electrode surfaces was determined using electrochemical impedance spectroscopy. The Nyquist plot representation of various electrodes, GC/NC and GC/NC@Chit was obtained in 1.0 M NaOH. The experiment was carried at a constant potential of 1.6 V (versus RHE), as shown in Fig. 11.

The fitting circuit that corresponds to the charge-transfer is shown in the inset of Fig. 11. To address the non-uniformity of the electrode surfaces, a constant phase element (CPE) is utilized in place of the capacitive component. The resistances Rs, Rc, and Q1 are commonly referred to as the solution resistance, charge transfer resistance, and constant phase element for layers. The computed fitting parameters are presented in Table 3. The charge transfer resistance of the GC/NC was measured to be 37 Ω cm^2^, whereas the GC/NC@Chit surfaces exhibited a lower resistance of 13 Ω cm^2^. The increased efficiency of oxygen evolution observed in GC/NC@Chit, as compared to pure GC/NC, can be attributed to the decrease of resistance’s values.

**Table 3:** EIS fitting parameters for Nyquist plot of different modified surfaces for OER.

Electrode	R_s_ (Ω cm^2^)	R_ct_ (Ω cm^2^)	Q	
			Y0 (F)	n
GC/NC	1.24	37	0.0001894	0.647
GC/NC@Chit	0.98	11	0.0003871	0.547

**Figure 12:**
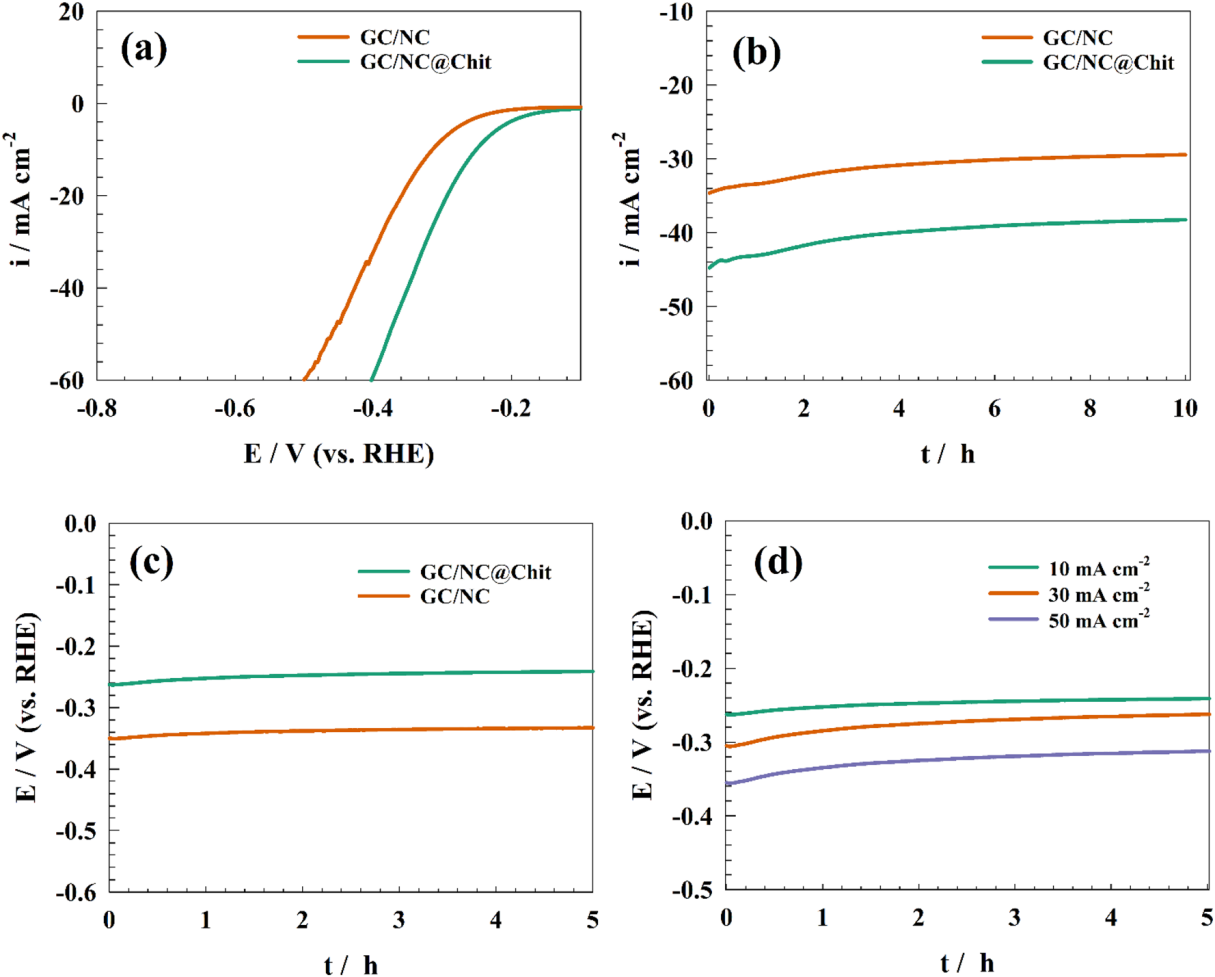
(**a**) Representation of LSV of the NC and NC@Chit for HER, (**b**) Chronopotentiogram of different modified surfaces for HER, (**c**) Chronopotentiogram of different modified surfaces for HER, (**d**) Chronopotentiogram of modified GC/NC@Chit at different constant current densities.

The study investigated HER on surfaces modified with NC and NC@Chit. Figure 12a shows the modified electrode's linear sweep voltammetry in a solution of 1.0 M NaOH.

The superior electrocatalytic performance of the modified GC/NC@Chit compared to the unmodified GC/NC can be attributed to the incorporation of chitosan into the electrocatalyst frameworks, enhancing both electronic and adsorption properties. The mechanism for HER in a medium with high alkalinity involves a reversible reaction between two molecules of water and two electrons, forming two adsorbed hydrogen atoms and two hydroxide ions. The Volmer step pertains to the dissociation of water. The corresponding Tafel step involves the reversible reaction between two hydrogen atoms adsorbed on a surface and forming a hydrogen molecule. However, the modified electrode had efficient HER at onset potential of − 0.26, − 0.2 V for NC and /NC@Chit surfaces, respectively. The current density reached 50 mA cm^−2^ at overpotential of − 0.48 and − 0.37 V for NC and NC@Chit surfaces, respectively.

Figure 12b illustrates the chronoamperogram of the GC/NC and GC/NC@Chit modified electrodes in a solution 1.0 M NaOH, with a consistent potential of − 0.3 V (versus the reversible hydrogen electrode, RHE). The measured current density exhibited a reduction of 14.3% and 12.6% for the GC/NC and GC/NC@Chit, respectively. The current decrease currently being observed is ascribed to the mechanical degradation of the electrocatalyst surface caused by the release of gases through said surface. The performance of our modified electrode in terms of hydrogen production was evaluated and compared with other catalyst and is tabulated in Table 4.

**Table. 4:** Comparative evaluation GC/NC@Chit electrode and other modified electrodes for HER.

Surface	ƞ (mV)@10 mA cm^–2^	Tafel slope (mV dec^–1^)	Electrolyte	References
GC/NC@Chit	− 240	71	1.0 M NaOH	This work
Co_2_FeO_4_@rGO	− 260	48	1.0 M KOH	^69^
Ni_2_Fe/N-doped porous C	− 198	83	1.0 M KOH	^70^
Ni/NiO core/shell nanosheets	− 145	43	1.0 M KOH	^71^
GC/Ag@CNT	− 570	148	0.5 M NaOH	^48^
NiFe-LDH/MXene/Ni foam	− 132	70	1.0 M KOH	^72^

However, slight deviations in the reduction current demonstrate the improved long-term durability of the electrodes in relation to the HER. Additionally, an investigation was conducted to assess the galvanostatic durability of the electrode for the HER. Figure 12c illustrates the application of constant potential chronopotentiometry at a consistent current density of 10 mA cm^−2^ across various modified surfaces, specifically GC/NC and GC/NC@Chit. Consequently, the electrode attained the potentials of − 0.33 and − 0.22 V, respectively. The decreased overpotential observed in the chitosan-based electrode can be attributed to its enhanced efficiency in facilitating the hydrogen evolution process. In addition, galvanostatic stability testing was conducted on modified GC/NC@Chit in a 1.0 M NaOH solution at various current densities, including 10, 30, and 50 mA cm^−2^. In contrast, the electrode achieved a current density of 50 mA cm^-2^ when the overpotential was measured at – 360 mV. (Refer to Fig. 12d).

**Figure 13:**
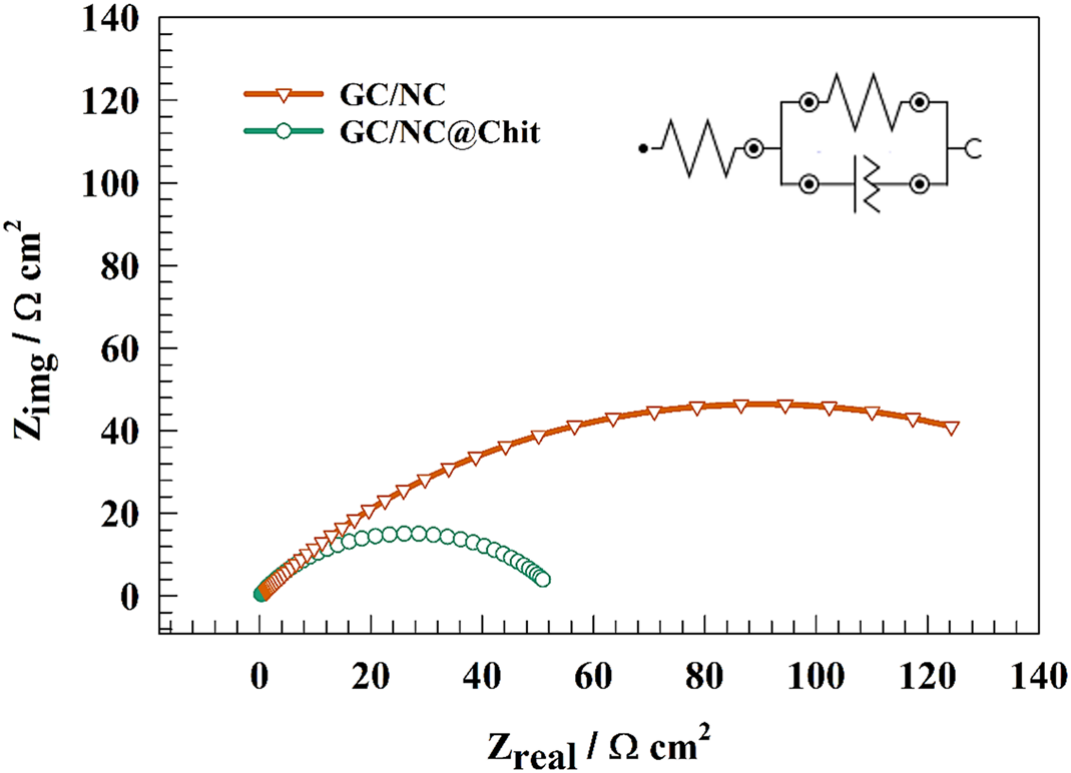
Representation of EIS plot of NC and NC@Chit surfaces at constant potential for HER.

EIS was used to confirm the charge transfer resistance across diverse electrode surfaces. As illustrated in Fig. 13, Nyquist representation for different surfaces of NC and NC@Chit, was acquired in a solution with a concentration of 1.0 M NaOH. The potential was kept constant at − 0.3 V (vs. RHE).

The corresponding fitting equivalent circuit is displayed in the inset of Fig. 13 with use of a constant phase element (CPE) as a replacement for the capacitive element in order to mitigate the non-homogeneity of the electrode surfaces. Table 5 displays the calculated fitting elements. The GC/NC@Chit material demonstrated a charge transfer resistance of 134 Ω cm^2^, whereas the GC/NC@Chit surfaces exhibited a resistance of 58 Ω cm^2^. The superior performance of GC/NC@Chit in hydrogen evolution as compared to pure GC/NC can be ascribed to the reduced charge transfer resistance.

**Table 5:** EIS fitting parameters for Nyquist plot of different modified surfaces for HER.

Electrode	R_s_ (Ω cm^2^)	R_ct_ (Ω cm^2^)	Q	
			Y_0_ (F)	n
GC/NC	3.4	134	0.0000786	0.513
GC/NC@Chit	4.45	58	0.0001224	0.568

**Figure 14:**
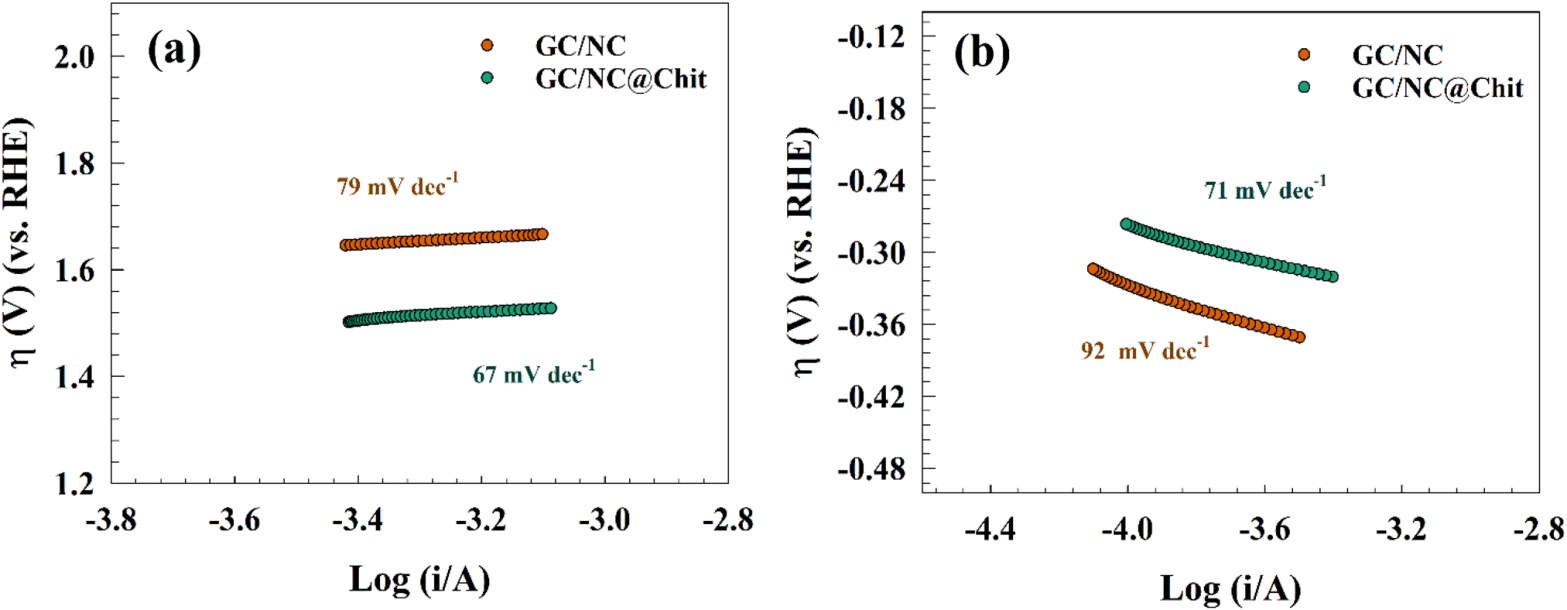
Tafel plot of NC and NC@Chit surfaces for (**a**) OER and (**b**) HER.

Furthermore, Tafel slopes are essential parameters in kinetic studies. In Fig. 14a, the Tafel slopes for OER and HER of modified GC/NC and GC/NC@Chit are depicted with values of 79 and 67 mV dec^−1^, respectively. The lower value for chitosan modified electrode indicated that the OER was favored over GC/NC@Chit compared with pristine GC/NC counterparts. The values of Tafel slopes were comparable with others reported in the literature for surfaces such as 136 mV dec^−1^ for NiO/C composite^73^ and 110 mV dec^−1^ for Fe(TCNQ)_2_/Fe^74^. Furthermore, Tafel provided slopes for HER are 92 and 71 mV dec^−1^ for GC/NC and GC/NC@Chit, respectively (see Fig. 14b). The values of Tafel slopes matched with those reported in literature like 135 mV dec^-1^ for Ni/NiO nanosheets^75^, 132 mV dec^−1^ for Co–S/CF^76^, and 148 mV dec^−1^ for Ag@CNT^48^.

## Conclusion

In conclusion, the synthesis of a composite material incorporating chitosan proved to be a successful strategy for enhancing the efficiency of both oxygen and hydrogen evolution. Comparative studies between pristine and chitosan-based NC revealed a synergistic effect, with the electrodes modified with GC/NC@Chit demonstrating notable efficacy in both capacitors and water-splitting applications. The pristine nickel cobaltite exhibited battery-like behavior, while the chitosan-based nickel cobaltite showed capacitor-like activity. Both modified electrodes showed high capacitance in an alkaline medium with provided values of 234 and 345 F g^−1^ (@1Ag^−1^). Additionally, high structure stability of spinel oxides characterized by repeated GCD represented high capacitance retention equaled 86, and 93% for NC and NC@Chit, respectively. The recorded current values were 50 mA cm^−2^ at 1.88 and − 0.36 V (vs. RHE) for OER and HER, respectively. The Tafel slope value ensured the water splitting favorability, whereas the lower value reflects the easier electron transfer process. Thus, the provided Tafel slopes at GC/NC@Chit are 67 and 71 mV dec^−1^ for OER and HER, respectively. Finally, the long-term stability reflected the good durability of GC/NC@Chit towards water splitting application.

## Data Availability

The datasets used and/or analyzed during the current study are available from the corresponding author on reasonable request.
